# New dosimetric guidelines for linear Boltzmann transport equations through comparative evaluation of stereotactic body radiation therapy for lung treatment planning

**DOI:** 10.1002/acm2.13464

**Published:** 2021-11-16

**Authors:** Matthew Webster, Sean Tanny, Neil Joyce, Amy Herman, Yuhchyau Chen, Michael Milano, Kenneth Usuki, Louis Constine, Deepinder Singh, Inhwan Yeo

**Affiliations:** ^1^ Department of Radiation Oncology University of Rochester Rochester New York USA

**Keywords:** Acuros XB, lung, planning guidelines, SBRT

## Abstract

**Purpose:**

To propose guidelines for lung stereotactic body radiation therapy (SBRT) when using Acuros XB (AXB) equivalent to the existing ones developed for convolution algorithms such as analytic anisotropic algorithm (AAA), considering the difference between the algorithms.

**Methods:**

A retrospective analysis was performed on 30 lung patients previously treated with SBRT. The original AAA plans, which were developed using dynamic conformal arcs, were recalculated and then renormalized for planning target volume (PTV) coverage using AXB. The recalculated and renormalized plans were compared to the original plans based on V100% and V90% PTV coverage, as well as V105%, conformality index, D_2cm_, Rx/D_max_, R50, and D_min_. These metrics were analyzed nominally and on variations according to RTOG and NRG guidelines. Based on the relative difference between each metric in the AAA and AXB plans, new guidelines were developed. The relative differences in our cohort were compared to previously documented AAA to AXB comparisons found in the literature.

**Results:**

AAA plans recalculated in AXB had a significant reduction in most dosimetric metrics. The most notable changes were in V100% (4%) and the conformality index (7.5%). To achieve equal PTV coverage, AXB required an average of 1.8% more monitor units (MU). This fits well with previously published data. Applying the new guidelines to the AXB plans significantly increased the number of minor violations with no change in major violations, making them comparable to those of the original AAA plans.

**Conclusion:**

The relative difference found between AAA and AXB for SBRT lung plans has been shown to be consistent with previous works. Based on these findings, new guidelines for lung SBRT are recommended when planning with AXB.

## 1 INTRODUCTION

Within the United States, non‐small cell lung cancer (NSCLC) is the leading cause of cancer death in both men and women, with an estimated 148,000 deaths in 2019.^1^ Early stage NSCLC had traditionally been surgically managed, but this was contraindicated for many patients due to comorbidities. To resolve this issue, surgery was replaced with conventionally fractionated radiation therapy. However, it was later found that this approach resulted in high rates of local failure and treatment‐related toxicities.^2^, ^3^

Over the last two decades, the use of stereotactic body radiation therapy (SBRT) has significantly developed.^4^, ^5^ This approach of using highly conformal, high dose, hypofractionated treatments has shown significant improvement in local control with minimal toxicity.^6^, ^7^ The use of SBRT for NSCLC is continuing to evolve with different treatment techniques, motion management, and fractionation schedules.

SBRT lung cases involve small fields being delivered through low‐density lung tissue. This causes an electronic disequilibrium effect near the air/tissue interfaces as the lateral range of secondary electrons becomes longer than the width of the small field segments.^8^ Thus, SBRT techniques demand highly accurate dose calculation algorithms in the media that involves tissue heterogeneity.

A recent major change in treatment planning for all sites is the introduction of Acuros XB (AXB) as an improvement on the previous analytic anisotropic algorithm (AAA).^9^, ^10^ The calculation model used in AAA is based on predetermined dose deposition kernels and is clinically acceptable for most situations, but loses accuracy in the conditions commonly encountered in SBRT lung treatment.^11^, ^12^, ^13^, ^14^, ^15^, ^16^, ^17^, ^18^, ^19^, ^20^ AXB uses a deterministic linear Boltzmann transport equation that expresses the interactions of various types of radiation in a given substance, which has been shown to be more accurate in the above conditions when compared to probabilistic Monte Carlo transport methods, the gold standard in dose deposition calculations.^21^, ^22^, ^23^, ^24^, ^25^ By increasing accuracy, the doses calculated with AXB deviate from those calculated with AAA. This raises an issue because the planning objectives of clinical trials for SBRT lung treatments have been developed using less accurate convolution–superposition and collapsed cone convolution algorithms, such as AAA. Therefore, it is critical to realize that established treatment planning guidelines may not be directly applicable to AXB plans. The higher accuracy of AXB necessitates a reevaluation of current SBRT lung treatment protocols.

It has been established that when planning with AXB, RTOG and similar guidelines can be met.^13^ It is unclear what this means in terms of radiation actually delivered. That is, provided that the same guidelines are met in AXB and AAA, the delivered dose from the treatment plans calculated with the two algorithms differs from each other, despite the planned dose being comparable. The dose coverage of the planning target volume (PTV) using AXB has been shown to drop up to 8% when compared to AAA plans, provided the same monitor units (MUs) determined using AAA are used.^9^ This means that AXB plans meeting the guidelines do not necessarily have the same biological effect (inferred by delivered dose) as plans created in AAA while matching the same dosimetric limits. It has been shown that when using MC to calculated plans, new dose guidelines are necessary.^12^

Our institution had previously treated SBRT patients using each algorithm to calculate the planned dose, initially using AAA and subsequently converting to AXB. Prior to the adoption of AXB within our clinic, SBRT lung cases were treated primarily with dynamic conformal arc (DCA) therapy with forward planning. However, when AXB was implemented, it was determined that this planning technique was insufficient to achieve the desired dosimetric qualities previously achievable when using AAA. By employing the more accurate AXB algorithm, the actual physical dose deposition within the patient went unchanged, but the plan quality worsened. This highlights the difference between the two algorithms and justifies a reevaluation of protocol values when assessing the quality of the plans. Our study evaluates current RTOG/NRG planning objectives and proposes modifications to make them more suitable for plans calculated using AXB.

## 2 METHODS

### 2.1 Treatment planning

A cohort of 30 patients treated in 2019 with plans calculated by AAA was retrospectively analyzed. During the CT simulation of all cases, patients were immobilized in a supine position on a GE LightSpeed CT scanner using a wing board with an index bar and a knee roll. The CT images were acquired with 512 × 512 pixels at 0.25‐cm slice spacing. Patients were simulated, starting with a slow CT, followed by a limited‐length 4D CT. Internal target volume (ITV) was contoured on maximum intensity projection image processed from 4D CT, and copied onto the slow CT for planning. PTV was created from a 5‐mm‐wide isotropic expansion of the ITV on the planning CT. The organs at risk such as contralateral lung, ipsilateral lung excluding the ITV, heart, and spinal cord were delineated.

All plans were first optimized to meet the region of interest guidelines (Chestwall: V30Gy < 30 cm^3^; Lung: V20Gy < 10%) and then evaluated for compliance with the other guidelines. The clinically used plans employed three or five fractions, ranging from 40 to 60Gy total dose with 60 Gy in five fractions being the most common fractionation. The different fractionation schemes do not affect the final results of this study because all suggested dose limits are relative to the total prescribed dose (Rx).

There is a large variety of possible patient geometries when dealing with SBRT lung cases. The size, location, and proximity to the chest wall or mediastinum all play a role in dose calculation and therefore could have a direct effect on the calculation differences between the two algorithms being evaluated. Therefore, a subgroup of 13 patients with island‐type tumors were evaluated separately. Island‐type tumors were defined in this study as the PTV boundary being at least 1 cm from the chest wall at its nearest point.

A 6 megavolt (MV) DCA‐based treatment technique was analyzed using several treatment planning guidelines. These were RTOG 0813, RTOG 0915, and NRG‐BR001 (NCT02206334) while varying in fractionation schemes. These all share similar dosimetric criteria for SBRT treatment of lung tumors, shown in Table 1. All plans were created using two or three DCA noncoplanar (NC) arcs. The typical arcs used can be seen in Table 2. The field aperture was set to conform to the PTV with a superior and inferior expansion of 1–2 mm, pending coverage. If necessary, these beams would be manually modulated by the treatment planner using forward planning to reduce hotspots in the PTV. The initial plans were then recalculated in AXB using the original MU and beam arrangements. The recalculated plans were subsequently renormalized to achieve 95% PTV coverage. Relative beam weights were not adjusted after the renormalization.

**TABLE 1:** PTV volume specific guidelines from RTOG and NRG guidelines for SBRT lung planning used in this study. Deviation values were interpolated based on PTV volume

PTV volume (cc)	Ratio of Rx isodose volume to the PTV (conformality index)		Ratio of 50% Rx isodose volume to the PTV, R50 deviation		Maximum dose (in % of Rx dose) 2cm from PTV in any direction, D_2cm_ (Gy) deviation	
	None	Minor	None	Minor	None	Minor
1.8	<1.2	1.2–1.5	<6	6–7.5	<50	50–57.0
3.8	<1.2	1.2–1.5	<6	6–6.5	<50	50–57.0
7.4	<1.2	1.2–1.5	<5	5–6	<50	50–58.0
13.2	<1.2	1.2–1.5	<5	5–5.8	<50	50–58.0
22	<1.2	1.2–1.5	<5	5–5.5	<54	54–63.0
34	<1.2	1.2–1.5	<4	4–5.3	<58	58–68.0
50	<1.2	1.2–1.5	<4	4–5	<62	62–77.0
70	<1.2	1.2–1.5	<4	4–4.8	<66	66–86.0
95	<1.2	1.2–1.5	<3	3–4.4	<70	70–89.0
126	<1.2	1.2–1.5	<3	3–4	<73	73–91.0
163	<1.2	1.2–1.5	<3	3–3.7	<77	77–94.0

**TABLE 2:** Gantry and couch angle of the typical DCA plans used

Beam (R/L)	Gantry (R/L) [deg]	Couch (R/L) [deg]
DCA	181–20/179–340	0
DCA	0–280/0–80	10/350
DCA	260–190/100–170	350 / 10

Plan recalculation was done to directly compare results between AAA and AXB calculations. Although the delivered doses are the same, the calculated doses were different. The renormalization of the plans allows for the evaluation of the two algorithms as they are clinically used. This allowed for the direct comparison of plans that were deemed acceptable using that algorithm's dose distribution.

### 2.2 Dose metrics

The recommended normal tissue dose limits, other than the guidelines mentioned in the previous section, are not explicitly reported here because they were a secondary priority to coverage, conformality, and dose falloff during planning. As a result, the analysis was focused on PTV coverage, dose conformality, and dose falloff. Target coverage was prescribed such that 95% of the PTV receives 100% of Rx (V100% = 95%; D95% = 100%). Both V100% and D95% were tracked. The PTV metrics utilized for the plans of this study were that 99% of the PTV should receive at least 90% of the prescribed dose (V90% ≥ 99%), the total volume outside the PTV receiving >105% of the prescription dose should be <15% of the PTV volume (V105% < 0.15 [%PTV]), and the ratio of the prescription dose to the 3D maximum dose (D_max_) must be ≥0.6 and ≤0.9. Conformality index (CI), taken as the ratio of the prescription isodose volume to the PTV volume, was utilized. The dose falloff was evaluated in terms of the ratio of 50% prescription isodose volume to the PTV volume (R50), and the maximum dose anywhere in the patient greater than 2 cm from the PTV in any direction (D_2 cm_). The last three metrics are volume specific and the exact deviation values of these last three criteria can be found in Table 1. For these volume‐specific guidelines, there are three possible results: no deviation, minor deviation, or major deviation. For all other criteria, there is only deviation or no deviation. Side‐by‐side plan comparison for statistical relevance was conducted using a double‐sided, paired *t*‐test.

For ease of comparison across different tumor sizes, D′_2cm_ is being reported, where

$$\;D{{}^{\prime }}_{2\mathrm{cm}}=\frac{D_{2\mathrm{cm}}\;(\mathrm{measured})}{D_{2\mathrm{cm}}\;(\mathrm{minor}\;\mathrm{deviation})}\;.$$

For example, for a treatment plan of a 10 cm^3^ PTV, a minor deviation occurs at 50%. If that plan had a calculated D_2cm_ of 48%, then the D′_2cm_ would be 0.96. A value below 1.0 meets the guidelines while a value of 1.0 or more indicates a deviation. Similarly, R50′ is the ratio of the R50 measured divided by the R50 minor deviation value. That is,

$$R50^{\prime }=\frac{R50\;(\mathrm{measured})}{R50\;\left(\mathrm{minor}\;\mathrm{deviation}\right)}.$$

In addition to the referenced guidelines, D_min_ to 0.03 cc of the PTV was recorded as a percentage of the prescription dose. D_min_ was measured because AXB is known to calculate more severe cold spots within the PTV when surrounded by lung tissue.^17^, ^18^, ^19^, ^20^ Because this is not a protocol objective, there are no associated deviation values.

To assess the impact of PTV size, the dose metrics being measured were compared to the target volume. A cutoff of 20 cm^3^ was used to deliniate between “small” and “large” PTVs. This cutoff was used to determine if there is a relative difference in the performace of AAA and AXB based on PTV size. For larger targets, the tissue inhomogeneity of the lung around the PTV may have a lessened effect.

### 2.3 Proposed guidelines

By comparing the baseline AAA plans with those recalculated in AXB, a ratio of the average value of each metric was obtained. The relative ratios between the metrics were then applied to each of the existing guidelines, to develop new guideline recommendations. The original planning guidelines did not take into account PTV location, despite the fact that it is known that these different patient geometries would have different physically delivered dose coverage.^14^, ^21^ Therefore, the new AXB‐based recommendations do not take geometry into account, assuming that the average values of the dose limits found using AAA are applicable to the average in AXB.

## 3 RESULTS

**FIGURE 1:**
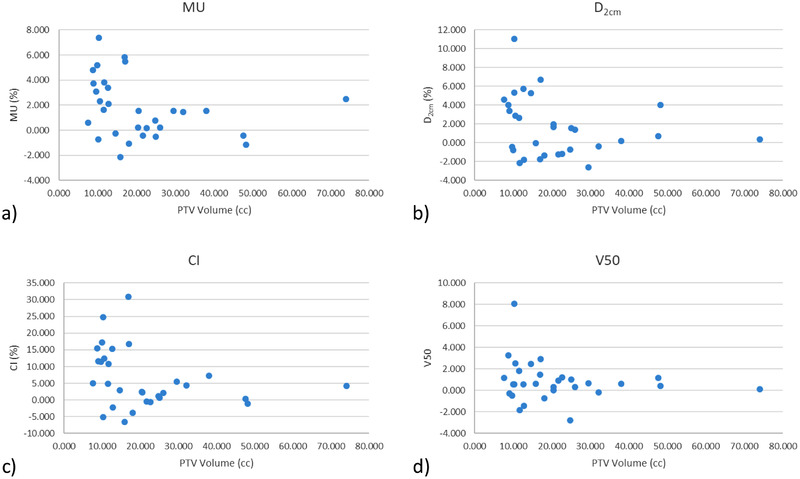
PTV volume dependence of (a) V50, (b) D_2cm_, and (c) CI recalculating AAA plans in AXB. When renormalized in AXB, the change in MU (d) also showed a PTV volume dependence. Targets under 20 cm^3^ showed more extreme discrepancies between AAA and AXB calculations

In the cohort studied, the PTV volume ranged from 7.58 to 74.06 cm^3^, with an average of 21.26 cm^3^. Table 3 presents the summarized dosimetry data from all 30 patient plans as well as the 13 island‐type target plans. Figure 1 shows relative difference between the initial and recalculated plans for D_2cm_, CI, and V50 as a function of PTV volume. It also shows the relative difference in MU needed to renormalize those same plans to achieve 95% coverage AXB. In each case, the vertical axis is (*M*_AXB_ – *M*_AAA_)/*M*_AAA_, where *M* is the given metric. Using the cutoff of 20 cm^3^, a statistical significance was found between PTV size and several dose metrics. These were MU, CI, and D_2cm_, whereas V50 had a *p*‐value of 0.14. This illustrates an underperformance of AAA for smaller targets. The other dose metrics studied did not show a significant dependence on PTV volume.

**TABLE 3:** Acuros versus AAA dosimetric comparison average over 30 total and 13 island‐type patients. V100%, V90%, and D95% are percent of prescribed dose. V105%, CI, D_2cm_, and R50 are percent of PTV volume. Statistically significant differences are marked in bold

Site	Nature	Calculation Model	D95% (PTV)	V100% (PTV)	D_min_ (PTV)	V90% (PTV)	Rx/D_max_	V105	CI	D'_2cm_	R50'	MU
All (*n* = 30)	Plan	AAA	100.00%	95.00%	90.5%	100%	0.830	6.3%	1.15	1.004	0.899	2115.0
	Recalculated	AXB	**98.21%** **(*p* < 0.01)**	**91.40%** **(*p* < 0.01)**	**88.4%** **(*p* < 0.01)**	**99.8%** **(*p* = 0.02)**	**0.823** **(*p* < 0.01)**	5.4% (*p* > 0.05)	**1.07** **(*p* < 0.01)**	**0.988** **(*p* < 0.01)**	**0.892** **(*p* = 0.03)**	2115.0
Renormalized	AXB	100.00%	95.00%	**89.9%** **(*p* = 0.05)**	100% (*p* > 0.05)	**0.810** **(*p* < 0.01)**	7.6% (*p* > 0.05)	1.15 (*p* > 0.05)	1.004 (*p* > 0.05)	**0.924** **(*p* < 0.01)**	**2154.3** **(*p* < 0.01)**
Site	Nature	Model	D95% (PTV)	V100% (PTV)	D_min_ (PTV)	V90%(PTV)	Rx/D_max_	V105	CI	D'_2cm_	R50'	MU
Island (*n* = 13)	Plan	AAA	100.00%	95.00%	90.4%	100%	0.817	3.8%	1.10	1.011	0.932	2301.0
	Recalculated	AXB	**97.03%** **(*p* < 0.01)**	**88.50%** **(*p* < 0.01)**	**87.4%** **(*p* < 0.01)**	**99.6%** **(*p* = 0.05)**	**0.810** **(*p* < 0.01)**	3.3% (*p* > 0.05)	**1.00** **(*p* = 0.01)**	0.999 (*p* > 0.05)	0.924 (*p* > 0.05)	2301.0
	Renormalized	AXB	100.00%	95.00%	89.9% (*p* > 0.05)	100% (*p* > 0.05)	0.787 **(*p* < 0.01)**	7.4% (*p* = 0.1)	**1.15** **(*p* = 0.03)**	1.029 (*p* > 0.05)	**0.984** **(*p* < 0.01)**	**2374.2** **(*p* < 0.01)**

### 3.1 Recalculation

As discussed in detail below, without renormalization, DCA plans generated with AAA and recalculated with AXB (noted this as AAA, AXB throughout this paper) had a decrease in all dosimetric values being evaluated. These effects can be seen in Table 3. Plans had a reduced V100%, dropping from 95% to 91.4%, and D95% fell from 100% to 98.21% (*p* < 0.01) overall. This is due to the fact that the calculated dose per MU is smaller with AXB than AAA on average because of the lack of buildup in lung media as modeled by AXB. As a result, across all cases (AAA, AXB), D_min_ (90.5%, 88.4%), V90% (100%, 99.8%), V105% (6.3%, 5.4%), CI (1.15, 1.07), D′_2cm_ (1.00, 0.99), and R50′ (0.899, 0.892) also decreased. The ratio of Rx/D_max_ decreased from 0.830 to 0.823. This corresponds to increase in the maximum point dose relative to the prescription dose of 1.5% in AXB plans in spite of the reduced PTV coverage. This agrees with the greater dose inhomogeneity of AXB calculations that have previously been found.^9^, ^10^, ^11^, ^12^, ^13^, ^14^, ^15^, ^16^, ^17^ All changes were statistically significant except V105% (*p* = 0.1). Island‐type tumor plans showed the same but more pronounced trends for most parameters due to their less‐buildup condition, compared with that of the other tumors that are closer to rib or chest wall structures. The more pronounced trend was shown, for example, by the reduced PTV coverage from 95% to 88.5% instead of 91.4% as stated above for the overall case. For D_2cm_ and R50, the trends were not statistically significant, potentially limited by sample size.

The recomputed plans showed fewer deviations from the protocol guidelines, as shown in Table 4. At the cost of PTV coverage, the total deviations dropped from 51 (49 major +2 minor) to 44 (42 + 2) overall and 21 + 1 to 19 + 1 for island‐type targets. More specifically, each deviation had equal or fewer occurrences after computing in AXB. Decreases were seen in CI (7, 6), D_2cm_ (18, 14), and R50 minor (24, 22). The relatively small change in the R50 minor was from the small change of R50′, from 0.899 to 0.892 with *p* = 0.03. Physically, this small change of R50′ was due to the 50% isodose line being largely in areas of charged particle equilibrium and being relatively robust against the algorithm changes unlike small areas with bigger changes in dose (e.g., a major change in dose may occur near a rib or target). These findings showed the performance of the recomputed plans by AXB against the guidelines that are more suited with AAA.

**TABLE 4:** Acuros versus AAA deviation comparison average for 30 total and 13 island‐type patients. Variances (v) and deviations (d) refer to RTOG and NRG protocol guidelines. Minor or major deviations with no occurrences are omitted

Site	Nature	Model	V105	CI	D_2cm_	R50	R50	Total
			Minor	Minor	Minor	Minor	Major	Minor, Major
All (*n* = 30)	Plan	AAA	0% (0)	23% (7)	60% (18)	80% (24)	7% (2)	49, 2
	Recalculated	AXB	0% (0)	20% (6)	47% (14)	73% (22)	7% (2)	42, 2
	Renormalized	AXB	0% (0)	27% (8)	50% (15)	60% (18)	23% (7)	41, 7
Site	Nature	Model	V105	CI	D_2cm_	R50	R50	Total
			Minor	Minor	Minor	Minor	Major	Minor, Major
Island (*n* = 13)	Plan	AAA	0% (0)	0% (0)	69% (9)	92% (12)	8% (1)	21, 1
	Recalculated	AXB	0% (0)	8% (1)	62% (8)	77% (10)	8% (1)	19, 1
	Renormalized	AXB	0% (0)	23% (3)	69% (9)	46% (6)	46% (6)	18, 6

The number of total minor deviations from V105%, CI, and D_2cm_ increased from 5 (0 + 1 + 4) to 29 (8 + 13 + 8) overall and from 4 to 11 for island cases with its trend in agreement with that of the case of DCA. The total number of deviations from R50 remained relatively constant overall (19 + 5 vs. 21 + 4) and island cases (8 + 4 vs. 9 + 3). This agreed with the finding for the DCA recalculation.

### 3.2 Renormalization

To renormalize the plans in AXB, the average MU increased from 2115.0 to 2154.3 overall, a 1.8% increase, as shown in Table 3. The change ranged from an increase of 7.4% to a decrease of 2.1%. There were seven cases where the MU minimally increased, typically less than 1%. In each of these cases, the PTV was situated directly next to a rib, which provided extra dose buildup (as much as 20% higher in AXB was reported in bone).^10^ After the renormalization, the plans became more similar to the original AAA plans. The differences (AAA, AXB) in V90% (100%, 100%), V105% (6.3%, 7.6%), CI (1.15, 1.15), and D′_2cm_ (1.00 to 1.00) were statistically insignificant for the overall cases. The difference in D_min_ (90.5%, 89.9%) remained significant. Also, the difference in Rx/D_max_ (0.830, 0.810; D_max_ = 120.5%, 123.5%) became more pronounced, corresponding to a higher D_max_. The difference in R50′ (0.899, 0.924) was also significant. This implies that when the same normalization was employed, AXB delivers a greater dose in healthy lung areas than AAA does. For island‐type tumors, these changes were more extreme. The MU change was from 2301.0 to 2374.2, a 3.0% increase. This is greater than the increase of the overall cases due to the greater distance of the island tumors from the chest wall and nearby ribs. More extreme changes were also found in the following values: V105% (3.8%, 7.4%), CI (1.1, 1.15), D′_2cm_ (1.01, 1.03), D_min_ (90.4%, 89.9%), and R50′ (0.932, 0.984). Only the changes in CI and R50′ were statistically significant.

After the renormalization, the total number of minor deviations from CI (8, 7), D_2cm_ (15, 18), and R50 minor (18, 24) in AXB was fewer than in AAA with a total of 41 in AXB and 49 in AAA. However, the decrease in R50 minor deviations is not indicative of better plan quality in AXB because most of the change comes from the minor deviation becoming major. The major deviations rose from two in AAA to seven in AXB. Most of them occurred in island‐type plans with one from the original AAA plans and six from the renormalized AXB plans. This comes from the reduction in the dose buildup in the PTV calculated by AXB when the PTV is surrounded by lung tissue. To achieve the same PTV coverage, the overall dose must be increased, causing the surrounding lower dose areas to receive a greater dose as well (i.e., MU increases). Overall, these trends mean that meeting planning guidelines, specifically R50, is more challenging when using AXB.

The current analysis may provide comparative understanding of the two algorithms in terms of their ability to provide dosimetry parameters in meeting the suggested guidelines. The preceding biology‐based analysis can provide dosimetry guidelines for AXB that are comparable to those for AAA.

### 3.3 Proposed guidelines

By taking the ratio of the recalculated versus initial planned doses from Table 3, the dose metrics were scaled, resulting in new guidelines. The new volume‐independent and volume‐dependent limits are shown in Tables 5 and 6, respectively. By comparing the baseline plans in AAA with those same plans recalculated in AXB, it can be seen that the D95% coverage dropped from 100% to 98.21%. Therefore, a dose reduction is recommended for SBRT lung patients being planned with AXB by 1.8% if equal coverage is desired. For a patient receiving 60 Gy, this would become 58.92 Gy. The exact magnitude of this change will be dependent on the size, shape, and location of the PTV. For example, island‐type tumors in this study decreased the coverage to 97.0%, which would correspond to 58.21 Gy. Note that with the exception of V105%, each term utilized for the ratio has statistical significance (*p* < 0.05). The proposed volume‐independent metrics, shown in Table 5, are V90% = 98.8%, V105% = 12.9%, and 59.5 < Rx/D_max_ < 89.2. The volume‐specific guidelines are shown in Table 6. As Figure 1 shows, there is a dependence on PTV volume for V50, D_2cm_, and CI. However, there is an insufficient sample size to give volume specific recommendations for all sizes of PTV volumes at this time. However, the majority of the plans studied (*n* = 29) had PTV volumes falling between 7.4 and 34 cm^3^. Therefore, new recommendations are given for this volume range. The largest change was seen in the CI, with minor deviations changing from 1.2 to 1.12 and major deviations going from 1.5 to 1.41. There was a relatively small difference between the old and proposed limits for V90%, 99.0% versus 98.8%, and range for Rx/Dmax, 0.60–0.90 versus 0.59–0.89. Therefore, keeping the old limits may be most prudent.

**TABLE 5:** Proposed new guidelines and current guidelines for volume independent metrics

Guideline	Old recommendation	Proposed recommendation
D95%	100.0%	98.2%
V100%	95.0%	91.4%
V90%	99.0%	98.8%
V105%	15.0%	12.9%
Rx/D_max_ (min)	0.60	0.59
Rx/D_max_ (max)	0.90	0.89

**TABLE 6:** Proposed new current guidelines and current guidelines for volumetric guidelines. Based on PTV volumes for this study, new guidelines are only proposed for PTVs between 13.2 and 50 cc. Other volumes would be expected to have similar trends

PTV Vol (cc)	Current CI variation	Current CI deviation	Proposed CI variation	Proposed CI deviation	Current D_2cm_ variation	Current D_2cm_ deviation	Proposed D_2cm_ variation	Proposed D_2cm_ deviation	Current R50 variation	Current R50 deviation	Proposed R50 variation	Proposed R50 deviation
13.2	1.2	1.5	1.12	1.41	50	58.0	49	57	4.7	5.8	4.66	5.75
22	1.2	1.5	1.12	1.41	54	63.0	53	62	4.5	5.5	4.46	5.45
34	1.2	1.5	1.12	1.41	58	68.0	57	67	4.3	5.3	4.26	5.25
50	1.2	1.5	1.12	1.41	62	77.0	61	76	4	5	3.96	4.96

These new guidelines with stricter values have been applied to the plans from this study. Based on these, new minor and major deviation counts have been applied to the recalculated and renormalized plans, as listed in Table 7. Compared to the current criteria, the AXB plans had an increase in all minor deviations. The largest change was seen in the CI, which rose from 6 to 11 in the recalculated plans and from 8 to 20 in the renormalized plans (Table 4 vs. 7). D_2cm_ minor deviations rose by 2 in both cases (14 to 16, 15 to 17) and the R50 had only one additional deviation in the recalculated plan (22 to 23).

**TABLE 7:** Evaluation of the AAA, recalculated, and renormalized plans when evaluated for protocol variations and deviations. AAA plans used the existing CI, D_2cm_, and R50 criteria, whereas the recalculated and renormalized AXB plans used the new proposed guidelines presented in this work

Site	Nature	Model	CI	D_2cm_	R50	R50	Total
			Minor	Minor	Minor	Major	Minor, Major
All (*n* = 30)	Plan	AAA	23% (7)	60% (18)	80% (24)	7% (2)	49, 2
	Recalculated	AXB	37% (11)	53% (16)	77% (23)	7% (2)	50, 2
	Renormalized	AXB	67% (20)	57% (17)	60% (18)	23% (7)	55, 7
Site	Recalculated	Model	CI	D_2cm_	R50	R50	Total
			Minor	Minor	Minor	Major	Minor, Major
Island (*n* = 13)	Plan	AAA	0% (0)	69% (9)	92% (12)	8% (1)	21, 1
	Recalculated	AXB	8% (1)	69% (9)	85% (11)	8% (1)	21, 1
	Renormalized	AXB	54% (7)	69% (9)	46% (6)	46% (6)	22, 6

After applying the new recommended dose limits to the recalculated AXB plans, the number of minor and major deviations compared to the original AAA plans became very similar (50, 2 vs. 49, 2) after significant improvement from (42, 2) against the RTOG and NRG limits. Comparing AXB with AAA plans under equal normalization against the new and existing guidelines, respectively, the AXB plans showed greater minor and major deviations (55, 7 vs. 49, 2), as shown in Table 7. This was contrary to the comparison when the current guidelines were used for both AXB and AAA plans (41, 7 vs. 49, 2), which showed a smaller number of minor deviations with AXB plans (41 vs. 55) with no difference in the number of major deviations (7 vs. 7). Because applying the proposed guidelines to AXB is equivalent to applying the existing guidelines to the AAA plans, they can be applied to the normalized AXB plans. For the AXB plans after the normalization, no re‐optimization was done after the new guidelines were applied. With the new target values, it is possible that some variations could be avoided if this was done. Therefore, the values in Table 7 are characteristic of the specific beam configuration used in this study.

## 4 DISCUSSION

Our results fit well into the literature surrounding the use of advanced dose calculation compared to convolution‐type algorithms, as shown in Table 8. The relative differences between AAA and AXB found in this study were comparable to other reported values from the literature.^10^, ^12^, ^13^, ^22^, ^26^ The averages in the table are weighted based on the number of patients from that study. Each of the studies used 6MV treatment beams, but implemented various beam configurations. The studies considered made use of DCA, volumetric modulated arc therapy (VMAT), NC static beams, and intensity‐modulated radiation therapy (IMRT) techniques. This allows for a sense of how AAA and AXB compare across different institutions and variety of treatment techniques. Rana et al.^13^ found similar trends as this study, with a direct comparison of AAA and AXB on 14 patient plans, noting that AXB plans demonstrated lower CI, R50, and D_2cm_ values. However, they did not evaluate the coverage, noting only that the lower CI indicated better conformality. Tsuruta et al. compared AXB and AAA against the fast‐photon Monte Carlo code XVMC using 26 patients and found statistically significant variations in PTV minimum and maximum doses.^10^ PTV D95% was not statistically significant for a difference between MC, AAA, and AXB. Ojala et al.^22^ found a correlation between tumor size and relative difference between AAA and AXB for D95%, D_2cm_, V30, and CI noting the greatest difference seen in PTV volumes under 25 cm3. This fits very well with the results shown in Figure 1. Krishna et al.^26^ found a statistical significance in D95% and D_max_ values when comparing AAA to AXB for IMRT cases. The other studies either did not report CI or used a different definition than from the guidelines, so it could not be directly compared for this study. Overall, our values fit well with the literature and support the need for updated AXB‐based planning guidelines. Given the good agreement between the data in this study compared to prior results, the proposed guidelines from this work are fairly robust. The exact values of such guidelines may warrant future work across more institutions.

**TABLE 8:** Dosimetric comparison of AAA to AXB from different studies. The average is weighted based on number of patients in the study. All results are given as (AAA – AXB)/AAA

Source	*N*	Technique (# of beams)	D95% (%)	D_max_ (%)	D_2cm_ (%)	R50 (%)	R100 (%)
Webster et al.	30	NC DCA (2–3)	–1.79	0.84	–1.59	–0.78	–3.79
Rana et al.	14	VMAT (2–4)	NA	2.25	–1.60	–1.15	–4.96
Ojala et al.	10	NC Static (5–9)	–5.00	NA	–0.80	–3.90	NA
Tsuruta et al.	26	NC Static (6–7)	–1.50	0.39	NA	NA	NA
Krishna et al.	15	IMRT	–0.85	1.13	NA	NA	NA
Average	NA	NA	–1.92	0.99	–1.45	–1.45	–4.16

Our results agree with the prior investigations, specifically for the island‐type patients in our cohort. Our investigation also noted changes in PTV coverage, when renormalization was done, between AAA and AXB, something other investigators have not evaluated. As discussed in the preceding paragraphs, we evaluated the performance of plans with AXB against the newly derived guidelines for AXB that are equivalent to the guidelines suited for AAA. This has not tried before.

Although the planning guidelines should be based on biological impact, in reality we typically use the usual existing guidelines, based on older and less accurate algorithms. This is true until a new set of guidelines that are equivalent to the previous guidelines are available for the new. This study has demonstrated that the renormalization, which we could adopt while using AXB, could mislead treatment planning by generating different numbers of variations for the cases of DCA (both overall and island), as shown in Table 4. Therefore, Tables 5 and 6 should be utilized if we were to meet the RTOG and NRG guidelines, based on their true delivered dose. Although the new guidelines are specific to the beam configuration of the DCA plans treated at our institutions, as their derivation utilized the ratios of various parameters in Table 3, their sensitivity to the technique is relatively low, as demonstrated in Table 8. As with the MU changes discussed previously, the dosimetric values that constitute the existing guidelines (Table 3) varied based on the physical location of the PTV (e.g., proximity to rib, island‐type). Although these results are calculated independently of PTV location, it would be informative to study patient outcomes based on PTV location within the lung and proximity to ribs.

The use of more advanced algorithms capable of reporting dose to medium requires other adjustments in the radiation physics workflow, specifically removing the 0.99 dose‐to‐muscle factor from machine calibrations. This factor had been recommended for use at centers enrolling patients on clinical trials. However, its use has been predicated on the previous generation of treatment planning algorithms that could only report dose to water. In centers using AXB for treatment planning reporting dose to medium, a recent AAPM Task Group Report 329 recommends removing the 0.99 dose‐to‐muscle factor from machine calibration as it would be double counting the dose conversion from water to muscle. Neglecting to remove this factor from machine output calibration, combined with our results demonstrating an increase in overall MU of 2% would lead to a net increase in dose delivery compared to dose delivered using a AAA calculated plan of 3%.

## 5 CONCLUSION

By switching over to the more accurate dose calculation algorithm of AXB from AAA, more MUs were needed for the equivalent calculated PTV coverage (V100% = 95%). This delivers greater doses to PTV and normal tissues, and makes it more difficult to achieve the RTOG and NRG protocol guidelines. These issues were especially apparent in island‐type tumors. As the guidelines are suited for kernel‐based algorithms such as AAA, new guidelines regarding acceptable dose to the PTV and healthy tissues (equivalent to the published guidelines) were developed for AXB, and evaluated for conformal arc planning techniques in this study. These new guidelines fit well with the existing literature directly comparing AAA to AXB for SBRT lung cases. They are recommended for lung SBRT treatments using AXB. A further study will include more clinical cases across different institutions and techniques with various geometrical characteristics of tumor in order to further fine‐tune the guidelines.

## CONFLICT OF INTEREST

The authors declare no conflict of interest.

## AUTHOR CONTRIBUTIONS

Matthew Webster, Inhwan Yeo and Sean Tanny were involved in conceptualiztion, investigation, methodology, writing the original draft preparation, and reviewing/editing; Matthew Webster and Sean Tanny were involved in data curation and visualization. Niel Joyce and Amy Herman were involved in clinical treatment planning, data curation and methodology. Drs. Chen, Milano, Usuki, and Singh provided patients for this study.
